# Hidden addiction: Television

**DOI:** 10.1556/JBA.2.2013.008

**Published:** 2013-06-14

**Authors:** Steve Sussman, Meghan B. Moran

**Affiliations:** ^1^Departments of Preventive Medicine and Psychology, University of Southern California, Los Angeles, CA, USA; ^2^School of Communication, San Diego State University, San Diego, CA, USA

**Keywords:** addiction, television, prevention, treatment

## Abstract

*Background and aims:* The most popular recreational pastime in the U.S. is television viewing. Some researchers have claimed that television may be addictive. We provide a review of the definition, etiology, prevention and treatment of the apparent phenomenon of television addiction. *Methods:* Selective review. *Results:* We provide a description of television (TV) addiction, including its negative consequences, assessment and potential etiology, considering neurobiological, cognitive and social/cultural factors. Next, we provide information on its prevention and treatment. *Discussion and conclusions:* We suggest that television addiction may function similarly to substance abuse disorders but a great deal more research is needed.

## Introduction

Recently, there has been consensus accumulating that there exist many types of behavior that might be considered addictions (e.g., see Sussman, Lisha & Griffiths, 2011). Addictions do not include only behaviors typically associated with excess and being a waste of potentially productive time (e.g., substance abuse, gambling). Addictions may include behaviors that may be intrinsically life-fulfilling but have appeared to spiral out of control (e.g., exercise, binge eating, relationships or work). Addictions also may include behaviors that are often intrinsically not associated with excess, are not generally considered life-fulfilling, but are often considered a waste of productive time (e.g., television viewing).

Sussman (2012) identified 16 categories of addictions based on an extensive electronic literature search of “types of addictions”. These categories were: Drugs, Food-related, Compulsive anti-social behavior (e.g., aggression), Technology/communications related (e.g., videogames, television), Gambling, Working, Social group-related (e.g., sex, love, platonic relationships), Physical attractiveness-focused (e.g., tanning, cosmetic surgery), Fantasizing (e.g., isolation, laziness), Exercise-related, Spiritual obsession, Pain seeking (e.g., self-mutilation, skin picking), Shopping, Thrill/adventure seeking, Hoarding (e.g., small collectables), and Voyeurism (e.g., celebrity or other idolization, gossiping). Television addiction is an example of a technology addiction, according to this scheme. Television (TV) addiction refers to out-of-control behavior pertaining to the medium of television,^1^^1^Given the prevalence of video content on the Internet, we include televised content accessed via the Internet in our definition of television. This includes content first broadcast on television (e.g., viewing an episode of “Modern Family” on Hulu) as well as content produced exclusively for the Internet (e.g., “House of Cards”, which can be viewed only via Netflix). However, for simplicity's sake we refer to this content as well as traditional televised content as “television”. We hesitate in applying this same label to many YouTube videos (which vary a great deal in air time, might be filmed and aired as well as viewed, and which has Web 2.0/social aspects). The latter might be more realistically placed under a more broad category of “screen addiction” (e.g., also encompasses videogames, social networking, and texting), which goes beyond the scope of this review. as opposed to a particular show on television (McIlwraith, Jacobvitz, Kubey & Alexander, 1991). That is, one may feel a subjective craving to view television a great deal to achieve a sense of satiation, become preoccupied with the idea of viewing television, not be able to predict how long one will watch TV (loss of control), and suffer negative life consequences as the result (Sussman & Sussman, 2011).

Widespread television viewing began in 1936 in Great Britain (http://www.teletronic.co.uk/tvera.htm; accessed December 14, 2012), and in 1947/8 in the United States (Barnouw, 1992). Prior to that time, before TV was widely available, there could not be a phenomenon of television addiction. However, the suggestion of the existence of television addiction began shortly after widespread viewing in the U.S. For example, Meerloo (1954), based on clinical case study observations, suggested that television addiction was a real entity, another source of “food for the senses” that might involve such a preoccupation so as to lead to generalized apathy, neglect of responsibilities, negativism, and fantasy. More recent work (1980s Swedish small general population sample) provided similar information, based on experience sampling data (Sjoberg & Magneberg, 2007). The first survey study on television addiction was conducted by Smith in 1982 (Smith, 1986), which led to questioning the prevalence of the phenomenon (though a majority of subjects perceived the existence of the phenomenon) but did identify a small sample of self-identified television addicts who viewed twice as much television as others.

Television viewing remains the most popular form of leisure activity in the United States (an average of 2.8 hours per day in the general population; U.S. Bureau of Labor Statistics, 2012), as well as in Australia and Western Europe (e.g., http://www.hsph.harvard.edu/obesity-prevention-source/obesity-causes/television-and-sedentary-behavior-and-obesity/; accessed January 30, 2013). Clearly, wide variation exists regarding what is considered heavy viewing, and the addictive aspect is more a function of interference with completion of life tasks rather than number of hours of viewing *per se* (Horvath, 2004). Because of the sheer amount of time people view TV, however, and because of the apparent consensus that TV addiction exists, this is an avenue of addiction research worth pursuing.

There has been argumentation and a little evidence for an opposing perspective regarding the impact of TV viewing, and the existence of TV addiction. For example, one discussion piece argued that television has been a scapegoat for social ills, derived in part from a television-as-drug metaphor (Mittell, 2000). Also, one study of college student subjects found that television viewing was inversely related to sensation seeking, alcohol, and drug use, and positively associated with religiosity, suggesting that it was an innocuous activity (Finn, 1992). Furthermore, television may facilitate relationship bonding (e.g., family gathering around the television). That is, television viewing can be a communal experience, as many television viewers come together to view and discuss programs (Andrejevic, 2008; Bagley, 2001). Finally, a considerable body of evidence indicates that television operates as a form of entertainment-education and is a source of health information for many viewers (see Singhal, Cody, Rogers & Sabido, 2004 for a review).

Thus, TV may exert positive as well as negative effects. However, just as alcohol use may serve a social lubrication function for some people and a source of drug dependence for others, so might TV operate differentially. Although TV addiction may occur in some people but not others (Appell, 1963; Krosnick, Anand & Hartl, 2003), television addiction is perceived as a reality among a majority of research study participants (though research subjects generally have been university students; McIlwraith, 1998).

The purpose of this paper is to attempt to summarize the work that has been completed on television as an addiction, including its qualification as an addiction and its assessment, etiology of television addiction, and its potential prevention and treatment. We consider whether or not television addiction might be considered a relatively safe substitute addiction (Sussman & Black, 2008), and conclude that there are several reasons that research in this arena is needed.

## Methods: Literature Review

To ascertain the state of research in this arena, we engaged in a literature search using the key words “television addiction” and “television dependence”. In Google Scholar there were 643 and 117 pages found, respectively (accessed August 22, 2012). In Ovid MedLine (1946-August Week 3 2012) there were only two pages, and zero pages found, respectively. Finally there were only nine pages, and three pages, located in PsycINFO. In total, we were only able to locate 33 published, relevant studies on the topic outside of books and dissertations. On the other hand, using the key words “television” and “consequences” we located 863,000 pages in Google Scholar. In the next section of this paper we briefly review examples of negative consequences of excessive TV viewing.

## Results

### Examples of negative consequences of TV viewing

Consequences mentioned in a cursory search of the literature include the impact of relatively heavy television viewing on (a) creating political or social biases (e.g., regarding presidential candidates, racial stereotyping) and shaping or increasing purchasing behavior (Jusoff & Sahimi, 2009; Romer, Jamieson & Aday, 2003), (b) increased aggression or fear of being victimized Romer, Jamieson & Aday, 2003),(c) attention and cognitive deficits (e.g., may contribute to Attention Deficit Hyperactivity Disorder; Christakis, Zimmerman, DiGiuseppe & McCarty, 2004; Sigman, 2007), (d) possibly a negative impact on academic achievement at least at extreme levels of viewing (though not in all studies; Paik, 2000), (e) predicting later cigarette smoking (Hancox, Milne & Poulton, 2004), (f) sleep difficulties (Sigman, 2007), (g) avoidance of relationship maintenance (Chory & Banfield, 2009), (h) lower life satisfaction (Frey, Benesch & Stutzer, 2007), (i) poorer body image among women (Grabe, Ward & Shibley Hyde, 2008), and (j) sedentary lifestyle leading to lower cardiorespiratory fitness, elevated serum cholesterol level and obesity (e.g., Anderson, Crespo, Bartlett, Cheskin & Pratt, 1998). Regarding the last consequence, for example, children 8–16 years old who watched four or more hours of TV per day were found to have greater body fat and a higher body mass index than those who watched less than two hours per day (Hancox et al., 2004; McIlwraith et al., 1991). One rather dramatic finding is that persons who watch six hours of television per day have been found to live 4.8 fewer years than lighter or non-viewers (Veerman et al., 2012).

### TV viewing as an addiction

The allure of television as an addiction not unlike drug misuse has infrequently been the topic of empirical study (Kubey & Csikszentmihalyi, 2002; McIlwraith, 1998). Yet, in several of the surveys that did measure this construct, prevalence of self-identified television addiction is approximately 10% in the United States (see Kubey & Csikszentmihalyi, 2002; McIlwraith, 1998). Furthermore, one study found that television addiction was significantly positively associated with addictions to alcohol, caffeine, cigarettes, chocolate, exercise, gambling and the Internet; and that the greatest “degree of addiction” was to exercise, caffeine, television, and alcohol (Greenberg, Lewis & Dodd, 1999). Prevalence of this addiction in the U.S. may be equivalent to the prevalence of addiction to alcohol or work, and only second to cigarette smoking (Sussman et al., 2011).

### TV addiction – Similarity/differences from substance use disorders, and its assessment

There are some similarities to but also some differences from the four criteria that compose substance abuse disorder in the DSM-IV. As with substance abuse disorder, role and social consequences can result (APA, 2000). First, one's ability to continue to function in roles at work or at home could become jeopardized as the result of one's television addiction, as case and ecological assessment studies suggest (Meerloo, 1954; Sjoberg & Magneberg, 2007). Regarding social consequences, heavy TV viewers are less likely to participate in community activities and sports (Kubey & Csikszentmihalyi, 2002), or focus on relationship maintenance communications, such as conflict management, positivity, or providing helpful advice (Chory & Banfield, 2009). Regarding legal problems or entering dangerous situations, it is difficult to see where TV addiction would apply (except for the rare situation in which people might view TV in their cars while driving). However, with advent of the DSM-5 (Jones, Gill & Ray, 2012) in May of 2013, legal consequences were dropped as part of the single Substance Use Disorder category. Craving replaced it as a criterion, and is associated with TV addiction (e.g., Kubey & Csikszentmihalyi, 1990; Meerlo, 1954; Sjoberg & Magneberg, 2007). Thus, TV addiction may fit DSM-5 type criteria rather well.

Likewise, as with the seven criteria that define Substance Dependence Disorder in the DSM-IV (APA, 2000), which are retained as part of the 11 criteria of Substance Use Disorder in the DSM-5, television addiction also exhibits consequential dependence-like features (e.g., Kubey & Csikszentmihalyi, 2002). There may be a need for *markedly increased amounts* of the behavior (increased time spent watching TV) to achieve the desired emotional effect, although there is not much empirical support for this criterion other than anecdotal reports or as considered within a heavy viewing factor (Horvath, 2004). There do appear to be subjective *urges to continue the behavior when one tries to stop* engaging in the behavior (e.g., anxiety and irritability when restrained from viewing TV, heartache and longing to watch TV, like drug withdrawal; Kubey & Csikszentmihalyi, 2002), there is evidence among problem viewers that the behavior is engaged in *over a longer period than was intended* (e.g., one may continue watching TV many hours rather than say 30 minutes, as planned), there appears to be a *persistent desire or unsuccessful efforts to cut down or control* the behavior, and it is feasible that a *great deal of time is spent on activities necessary to begin or continue the behavior, or recover from its effects* (e.g., one may try to work at home as often as possible to be able to watch TV, one may frequent restaurants or bars that have TVs in them; later one may have to catch up on work).

In addition, *important social, occupational, or recreational activities are given up or reduced* because of the behavior (e.g., one may reduce engagement in some prosocial hobbies, or ignore job or family duties, while watching TV; McIlwraith, 1998), and the *behavior may continue despite knowledge of having a persistent or recurrent physical or psychological problem* that is likely to have been caused or worsened by the behavior (e.g., one may suffer from relative social isolation, financial loss or weight gain as a result of excessive TV viewing, yet continue to watch TV excessively).

Horvath (2004) created a 35-item measure of TV dependence, composed of seven sets of five items intending to tap each of the seven dependence criteria. Factor analysis indicated that 31 of the items loaded on four factors among a sample of 300 diverse subjects. These factors were: problem viewing (e.g., “alienating my loved ones”, “feel bad but can't stop”, “all my leisure time”), heavy viewing (e.g., “longer time than intended”, “think I should cut down”, “guilt about watching so much”), craving for viewing (e.g., “watch more to feel the same”, “can't reduce amount”, “same amount, less satisfaction”), and withdrawal (e.g., “could easily go without it”, “can't imagine going without”, “withdrawal when unable to watch television”). She also developed a shorter, dependence CAGE-like measure (“Have you ever felt you ought to Cut down on the amount of television you watch?”, “Have people Annoyed you by criticizing your television watching?”, “Have you ever felt bad or Guilty about your television watching?”, and [as an Eye opener] “Do you usually turn on the television first thing in the morning?”).

In summary, TV addiction has several similarities and some differences from substance abuse and dependence disorders. Legal consequences are unlikely, as are immediate physical dangers. Thus, it may be a highly prevalent but socially tolerated problem that can be adequately assessed. However, otherwise, this type of behavior appears to meet the other criteria typical of the addictions (Sussman et al., 2011), and there are numerous potential negative consequences that may result from heavy or addictive viewing. Its etiology, prevention, and treatment are worth consideration, and are discussed next.

### Etiology of television addiction

Since the whole concept of TV addiction is not well-researched, it is not surprising that the etiology of television addiction is not well understood. As with other addictive behaviors, one may speculate that television addiction stems from aberrations in neurobiological and social learning processes. For example, TV addiction might be expected particularly among persons experiencing inadequate turnover of mesolimbic dopamine, who are relatively anxious or bored, who have difficulties with social communication, or who are consistently reinforced for spending time watching TV, as is the case with several other addictions (e.g., Freimuth et al., 2008; Sussman et al., 2011). Indeed, McIlwraith (1998) found, among a subsample of self-identified TV addicts, relatively high reports of anxiety, being more likely to use TV to distract them from unpleasant thoughts, and being more easily bored and distracted, than others in the sample.

Some communications researchers argue that enjoyment of media use is the equivalent of need satisfaction (e.g., Tamborini, Bowman, Eden, Grizzard & Organ, 2010; Tamborini et al., 2011). In other words, pleasure a person derives from television viewing may be attributable to that medium's satisfaction of the viewer's hedonic and non-hedonic needs, from which addiction may stem (Sussman, 2012).

In particular, two lines of theory and research within the communications discipline (Media Systems Dependency Theory and Uses and Gratifications Theory) provide classifications of motivations for television viewing or outcomes of viewing that may be helpful in conceptualizing the etiology of television addiction. According to Media Systems Dependency Theory (MSD: Ball-Rokeach, 1985, 1998; Ball-Rokeach & DeFleur, 1976), individuals use media more heavily in times of ontological insecurity. In other words, when an individual feels insecure in his or her identity, relationships or environment, he or she becomes more reliant on media. Specifically, there are six main goals individuals may seek when using media: self- and social understanding, action and interaction orientation, and solitary and social play. Self-understanding is the extent to which an individual feels secure in his or her beliefs, values and identity. Social understanding refers to the extent to which an individual has “knowledge ... of how society and its institutions function” and his or her role in that society (Loges, 1994). Action orientation refers to the knowledge of how to behave appropriately in given situations, while interaction orientation has to do with the acquisition of interpersonal skills. Finally, solitary and social play refers to escape, entertainment or diversion – either alone or with others – which one can obtain through media use. Consistent with MSD Theory, TV addiction is relatively likely to occur when one feels insecure in identity, feels alienated socially, feels unable to act or learn to act appropriately in social contexts, and is preoccupied with TV viewing as a means of solitary and social play.

Similarly, the Uses and Gratifications Theory (U&G: Katz, Blumler & Gurevitch, 1973) assumes that television viewers are active individuals who choose to watch television in order to fulfill specific needs (e.g., to obtain a gratification). These gratifications can be classified as: diversion (including escape and release), personal relationships (using television to facilitate relationships or as a substitute for them), personal identity (understanding and reinforcing one's values, attitudes, etc.) and surveillance (understanding and keeping track of what is going on in one's world; McQuail, Blumler & Brown, 1972). Watching TV to escape, as a substitute for social play, or as a means of reinforcing one's values certainly would seem able to facilitate addictive viewing.

Both the MSD and the U&G theories focus on functional aspects of television viewing. Although each theory stems from a different set of assumptions (see Ball-Rokeach, 1998), their typologies of gratifications or goals sought from television viewing contain similarities. Specifically, using these theories, motivations for television can be conceptualized as: (a) learning (e.g., understanding or orienting oneself to one's world), (b) connection (with either real or fictional people) and (c) affect regulation (via diversion or entertainment). Each of these motivations provides insight as to the potential etiology of television addiction.

First, a “learning” motivation can lead individuals to search TV for information needed to function in their world, thus providing them with a sense of security. Viewing political news channels, for example, can help a viewer decide which candidates to vote for. Additionally, information learned through television viewing may provide individuals with functional rewards. For example, many programs – most notably talk shows (e.g., Dr. Oz) – provide individuals with recommendations for products and behaviors that they can use to improve their lives. Importantly, structural features of television technology that impact the way in which information provided is processed (passive involvement, fast pacing), may make television viewing more entrancing, information provided seem more believable, and contribute to making this medium addictive (Kubey & Csikszentmihalyi, 2002).

Television can also be used to compensate for a paucity of interpersonal “connections”. For example, some TV shows or large scale cultural events (e.g., “The Simpsons”, “Superbowl”) that emphasize the family gathered around the TV, or the involvement of many people focused on an event, may assert the primacy of TV viewing as a focal point for social bonding or recreation, even when one is watching TV alone. In fact, some research has shown that individuals who are lonely tend to watch more television (Perse & Rubin, 1990) and, building on this research finding, Wang, Fink and Cai (2008) found that men who are chronically lonely are more likely to develop parasocial (imagined) relationships with media figures than are their non-lonely counterparts, perhaps as a way to compensate for this loneliness. It is likely that a “social connection” motive may facilitate preoccupation with TV viewing and subsequent addiction.

Regarding “affect motives” for watching TV, Kubey and Csikszentmihalyi (2002) summarized their work with the Experience Sampling Method (ESM) of time sampling daily life experiences and noted that people feel relaxed (quickly) when watching television. However, they feel less relaxed right after terminating television viewing, along with more difficulty concentrating, and with continued lower alertness. As such, television can be used to “regulate” one's level of arousal and mood. McIlwraith et al. (1991) suggested that television may modulate arousal level (to reduce neuro-biologically-based overstimulation). Thus, persons who are relatively overstimulated may gravitate to television viewing to modulate arousal level. Conversely, individuals who are seeking arousal may use television as a way to obtain that stimulation (Bryant & Zillmann, 1984). Also, Anderson et al. found that experience of stressful life events is associated with relatively greater time spent viewing television, suggestive of stress-reduction motives often attributed to addiction (Anderson, Collins, Schmitt & Jacobvitz, 1996). TV viewing's potential ability to temporarily satiate various affect-based motives may qualify it as a potential object of addiction (Sussman, 2012; Sussman & Sussman, 2011).

It must be noted that use of television for learning, connection or affect-based motives is not inherently problematic and in many cases is beneficial (e.g., learning healthy lifestyle tips from a talk show or staying on top of a popular series in order to bond with co-workers). However, the fact that TV has the capacity to satiate these needs may predis-pose it to be a source of problematic use for vulnerable individuals. Certainly, much more etiology research is needed to confirm or disconfirm these speculations but few empirical studies have been completed in this arena thus far, and are needed.

### Prevention and treatment

There is very little empirical work on the prevention or treatment of television addiction. The next two subsections examine potential prevention and treatment strategies.

### Prevention

Whether television is harmful is a function of what programming is being viewed (e.g., violence on TV), who is viewing (e.g., addiction prone), how often TV is viewed (e.g., more than two hours per day may become a problem), and whether viewing involves other significant others (which may or may not be relationship enhancing; Kubey, 1990). Parents can assist in making their children media literate and screen out certain types of programming (Jusoff & Sahimi, 2009; Singer & Singer, 1998). Media literacy could be provided through a variety of modalities (e.g., schools), and involves a deliberate analysis of media programming. In such an analysis, depending on the age of the viewer, one may be asked to search contents for advertising ploys, stereotypes, major themes and purposes of a show, whether contents are attempting to be realistic or a fantasy, and how shows might impact ones feelings (Singer & Singer, 1998). Several impactful media literacy curricula have been implemented over the last 20 years (Singer & Singer, 1998), and these could focus more on limiting the amount of television viewed as well as interpretation of programming.

Certainly, counseling that attempts to facilitate a secure attachment style with others may help delimit reliance on television as a form of passive social contact. Clients also might be engaged in cognitive restructuring to remove fantasy-based thinking (e.g., if there was over-identification with television characters) that could lead to later television addictive behavior. Mood management techniques might be instructed to reduce the desire to search out external sources of relief such as the TV.

Additional types of prevention strategies might be envisioned. For example, since it is possible that development of television addiction may occur through social learning processes (e.g., exposure to the behavior of significant others, mass media influences), corrective information about healthy and unhealthy television viewing patterns may be instructed as part of school health curricula, or through other types of community health promotion programming. One may, for example, be presented with a set of age-appropriate/inappropriate television viewing scenarios. One could be guided through a decision-making sequence about the benefits and costs to self and others for each scenario, and learn when costs outweigh benefits.

Prevention efforts can also take advantage of the fact that the source of addiction (television) is also a communication medium. Specifically, ads could be run on television to alert individuals as to the signs of dysfunctional television viewing and provide information on how to seek assistance prior to developing a deeply ingrained addiction. Prevention efforts could also take advantage of entertainment-education strategies, whereby educational storylines are embedded within a television program, that attempt to limit number of hours of viewing or unhealthy viewing motivations.

One may also consider policy actions that could serve a preventive effect. For example, warning statements might be placed on television consumer channel packages/plans to encourage limiting viewing time to no more than two hours per day. Certainly, some persons might view such action as extreme. However, if there is evidence that the TV media promote addiction, and if TV addiction prevalence is sizable (e.g., 5% or greater of the population), then such action would seem justified. There is much research needed regarding types of prevention programming that could be developed.

### Treatment

Some work exists regarding the *treatment* of television addiction, though most of this work is clinical (non-research). Kubey and Csikszentmihalyi (2002) suggest that TV viewers keep a diary for a few days to identify the amount of time TV was being viewed and how much the viewer benefited from the various programs. In addition, they suggested that families might promote other activities (e.g., involving live social interactions), exercise willpower or enforce time limits, make use of channel blocking features, plan which shows to watch ahead of time (decision making), limit the number of TVs in the home, limit location of TVs, and otherwise learn mindful television viewing (e.g., through media education).

There does not appear to be a highly organized 12-step program on recovery from television addiction, though there are some blogs (in particular, see TVAA; http://tvaa.blogspot.com/; accessed on December 14, 2012). Therapy for television addiction (outside of 12-step organizations) appears in the literature, and also may be helpful. Various individual-level therapy options might be considered. Motivational Interviewing (MI) may help TV addicts understand maladaptive functions of excessive viewing. For example, one may learn through MI techniques that their TV viewing involves an ongoing pattern of equivocation about issues of trust and intimacy. One may then try to reduce the discrepant feelings by deciding to practice entering relationships, particularly in locations where television is not available.

Developing non-TV hobbies may be important. The therapist may establish short-term goals with a TV addict that could include signing up for community courses (e.g., photography), participation in meditation or exercise, and making friends. Group therapy also may be a helpful option. One may conjecture that group therapy techniques (e.g., use of psychodrama) may help one decrease illusions toward television, and help one understand ones feelings toward long-term significant others such as one's nuclear family. One may also learn through group interaction how to better participate in healthy relationships, which may be less immediately engrossing but more rewarding in the long run.

## Conclusions and Future Research

Television addiction indicates a constricted pattern of repetitive behavior directed toward one activity that leads to negative role or social consequences, which also has several dependence-like features making it difficult to stop. It may involve brain neurotransmission processes similar to the effects of drug misuse, and may be a substitute addiction for drug misuse for some persons. This arena has been studied primarily through use of clinical inference in books and communications theoretical articles. Very little empirical (data-driven) work has been completed. Future work should investigate the social, psychological and physiological responses to television. In particular, we need to learn if there is a quantifiable threshold for TV viewing that we might identify as addiction. We need more research that identifies harm from addictive TV viewing specifically. Empirical studies that investigate neurobiological impacts (e.g., brain pattern changes, neurochemical production changes), would be an excellent direction to take and draw parallels to other addictions on which such data is available (e.g., drug abuse, gambling).

Television addiction is a useful area to study for several reasons. First, if it is true that 5% to 10% of the population suffers from television addiction, its prevalence alone would be a cause of concern that needs remedy. Further validation of this estimate is needed. With the options of TV screen involvement ever increasing (e.g., as accessed on the Internet as well as television set) it would not be surprising if the prevalence of TV addiction is increasing. Second, it is possible that it is an addictive process that arises during childhood or adolescence as with other problem behaviors, such as tobacco use or drug experimentation. Importantly, it is feasible that TV addiction is one of the first to be present among people as children. They may learn that they can regulate affect through a behavior or substance at a young age. This addiction, or replacement addictions after an addictive process becomes engrained via TV, may exert a life span hold on vulnerable persons (Sussman, 2013).

Third, and related to the second reason, the functional overlap between substance misuse and television addiction is striking in some ways, as previously discussed. In investigating both disorders concurrently, one may be able to learn about the underlying processes involved (e.g., impact on mesolimbic dopaminergic turnover). Fourth, some researchers and practitioners might consider TV addiction as a relatively safe substitute for drug or other addictions. Drug recovery centers typically have television rooms in which a great deal of viewing time is going on. One may conjecture that, while being a sedentary and potentially wasteful activity, at least one is not likely to incur legal or immediately physically destructive consequences (e.g., overdoses or accidents) likely to be experienced via drug abuse. Future research is needed to explore this possibility.

Finally, the mere lack of research completed deters one from achieving conceptual clarity regarding television addition. It may overlap with personality or other disorders such as histrionic or narcissistic personality disorders, or other behavioral addictions, which needs to be delineated. That is, the existence of TV addiction as a “stand-alone” phenomenon still needs investigation.

With many addictions, there is the notion that an individual is engaging in the behavior “too much”. That is, the person is losing money (e.g., spending a lot of money to buy alcohol, or on paying for consequences related to impulsive behavior resulting from being drunk), risking physical consequences (e.g., alcoholic liver disease), or is experiencing a diminished scope of activity (e.g., passing out, or passive experience of life, due to being drunk all the time), through over-investment in the addictive behavior. For example, there is some consensus that drinking more than two drinks of alcohol a day is physically hazardous, and that drinking more than six drinks a day may be considered alcoholic drinking (e.g., see http://www.icap.org/LinkClick.aspx?fileticket=KtXj8PGibT8%3D&tabid=75; accessed February 8, 2013; Sussman & Ames, 2008). However, it may be relatively difficult to determine an addictive level of TV viewing frequency, particularly because certain forms of television have been shown to have positive consequences for viewers (e.g., entertainment-education: Singhal et al., 2004). It is possible that an equivalent amount of viewing time may be addictive (cause problems) for some people but not others, depending on competing life demands. Trying to establish a consistent normative threshold of addictive viewing time may be impossible. For example, among U.S. university students back in 1998, self-identified TV addicts were found to watch TV an average of about 21 hours a week, whereas those who did not so identify watched an average of approximately 13 hours per week (McIlwraith, 1998). However, that amount of TV viewing reported by those TV addicts equates to three hours per day, about the same as the average viewer reported in the recent Bureau of Labor Statistics time use survey for the general population (U.S. Bureau of Labor Statistics, 2012). Strangely enough, Neilsen put out a report in 2009 that indicated an average viewing level of 141 hours per month (almost five hours a day) among people who own a TV (http://blog.nielsen.com/nielsenwire/wp-content/uploads/2009/09/ThreeScreenReport_US_2Q09REV.pdf; accessed December 14, 2012). Thus, it will take some research effort to better explore how viewing time relates to addictive viewing, versus other parameters of addictive viewing (e.g., ignoring other activity demands, self-perceptions of one's TV viewing, impact on physical health).

Notably, the media landscape has changed considerably since 1998. The boundaries of television viewing are expanding. For example, many people are watching television shows online via Hulu or Netflix, are watching timeshifted TV, or are even viewing TV on mobile phones (http://blog.nielsen.com/nielsenwire/wp-content/uploads/2009/09/ThreeScreenReport_US_2Q09REV.pdf; accessed December 14, 2012). Gauging total television viewing time may need to be revised, and contexts of heavy television viewing may shift. Social normative confusion over the boundaries of healthy television viewing suggests there is much to be learned. Related to this are the numerous other forms of mediated content to which individuals are exposed, such as YouTube videos which allow viewers to post comments and interact with other viewers, movies and video or computer games. All of these media might be considered as a constituent of a broader category of “screen addiction” in future research. It is possible that, while similar to television, these forms of content may fulfill different motivations. Alternately, these forms of content may more fully satisfy a need addressed by television use. For example, YouTube allows viewers to interact directly with each other by posting comments, posting videos to one's social media pages (Facebook, Twitter, etc.) and directly emailing or texting videos to one's friends. Thus, this form of video content may be particularly well-suited for fulfilling a social connection need, and may be addictive. Clearly, more research is needed to delineate the positive and negative effects of these different forms of video content (e.g., see Kuss & Griffiths, 2011, regarding social network addiction).

In summary, there does appear to be a phenomenon of television addiction, at least for some people. TV addicts are likely watch TV to satiate certain appetitive motives, demonstrate preoccupation with TV, report lacking control over their TV viewing, and experience various role, social, or even secondary physical (sedentary lifestyle) consequences due to their out of control viewing. These consequences are in part contextually driven, due to amount of viewing time contrasted with competing time demands. As with other addictions, it is likely that self-reported TV addicts suffer from multiple addictions (Sussman et al., 2011), and that both prevention and treatment approaches may be needed for them. Much research is needed to better understand this addiction which *prima facie* seems relatively innocuous but in reality may incur numerous life problems.
